# Lugol's iodine-guided excision margin determination in oral mucosal lesion management

**DOI:** 10.6026/973206300212631

**Published:** 2025-08-31

**Authors:** Ranjeeta Yumnam, Shruthi Rangaswamy, Deepak Ningombam Singh, Jenny Atom, Ahanthem Nandita, Sachidananda Chungkham

**Affiliations:** 1 Department of Oral & Maxillofacial Surgery, Dental College, Regional Institute of Medical Sciences, Imphal, Manipur, India; 2 Department of Oral & Maxillofacial Surgery, Rajarajeshwari Dental College, Bangalore, Karnataka, India; 3 Department of Oral Medicine and Radiology, Dental College, Regional Institute of Medical Sciences, Imphal, Manipur, India; 4 Department of Conservative Dentistry & Endodontics, Dental College, Regional Institute of Medical Sciences, Imphal, Manipur, India; 5 Department of Periodontics, Dental College, Regional Institute of Medical Sciences, Imphal, Manipur, India

**Keywords:** Diagnostic efficiency, dysplasia, excision margins, lugol's iodine, oral mucosal lesions

## Abstract

Lugol's iodine (LI) is recognized for its diagnostic efficacy in identifying and delineating sufficient margins of the biopsy region. Therefore, it is of interest to evaluate the variance in excision margins between visual assessment and LI as well as to histopathologically analyze the disease-free margin post-excision. The diameters of the lesions were statistically compared before and after the application of LI in 18 male patients. The histopathological report was assessed for disease-free margins. Data shows that LI stain is an essential method for assessing the progression/excision margins of suspicious oral lesions, hence aiding in the early identification of oral cancer.

## Background:

The prevalence of oral cancer (OC) varies significantly across different regions globally, ranging from 2 to 10 cases per 100,000 individuals annually [1]. India, Sri Lanka, Pakistan, and Bangladesh are among the South Asian nations with the highest prevalence [2]. While 95% of OCs manifest in individuals over 40 years of age, there appears to be an increasing rate of OC among those under 40. The report pertains to the prevalence of high-risk behaviours, including both alcohol and tobacco consumption, among teens and young adults. Most oral squamous cell carcinomas (OSCC) arise from oral potentially malignant disorders (OPMDs). Accurate diagnosis and timely intervention for oral potentially malignant disorders may decrease the risk of malignant transformation in oral lesions [3]. The best strategy to lower the death, morbidity and deformity from OC is thought to be early detection, when lesions are small or localised [4]. Visual inspection remains the benchmark for identifying early epithelial alterations. Nonetheless, the basic visual assessment is widely recognised to be constrained by interpretation that is subjective and the possible, albeit infrequent, emergences of dysplasia along with early OSCC in regions of apparently sound oral mucosa. Consequently, supplementary strategies have been proposed to enhance our capacity to distinguish between benign anomalies and dysplastic alterations, and also to detect dysplastic regions that are not discernible to the human eye [5]. Various strategies have been devised to enhance clinical evaluation and refine the detection of early OC. Lugol's iodine (LI) is said to possess diagnostic efficacy in identifying and delineating sufficient margins of the biopsy area. The basis of LI staining involves the reaction between iodine and glycogen in the cytoplasm, resulting in a colour shift described as the iodine-starch interaction. The amount of glycogen in tissue is negatively correlated with the extent of keratinisation, as glycogen is essential in the keratinisation process. The lack of cellular differentiation and increased glycolysis in malignant cells impede the iodine-starch interaction. In mucosal evaluation, the use of LI on suspected lesions indicates that normal mucous membranes display a dark or mahogany coloration due to elevated glycogen levels, while dysplastic tissue remains unstained and appears pale compared to adjacent tissue. The LI solution, known as Schiller's test, serves as a diagnostic tool for identifying lesions of concern in the oesophagus, gastrointestinal tract, and gynaecological areas during endoscopic and colposcopic procedures [6].

The efficacy of LI staining in the oral cavity is limited to non-keratinized mucosa; alternative approaches should be utilized for identifying early cancer and delineating borders in other mucosal areas. Regions of the mouth with significant keratinisation, such as the connected gingiva and hard palate, fail to readily absorb LI [7]. The early identification of OSCC continues to be a critical concern for healthcare providers and patients alike. Vital staining techniques, such as toluidine blue and LI solution are frequently utilized in the identification of OSCC; however, there is limited data regarding the latter. LI in preliminary screening can be utilized to delineate the boundaries and extent of oral lesions. Vital staining is a recognized method for identifying non-visible, non-palpable, abnormal premalignant regions of the oral mucosa. The term oral intraepithelial dysplasia is pertinent for both the "invisible" at-risk regions and the observable lesions of concern within the oral cavity and oropharynx [8]. The LI staining technique is highly effective for delineating boundaries and assessing the extent of dysplastic changes in suspicious lesions, contingent upon the application of the LI solution. Therefore, it is of interest to ascertain the disparity in excision margins between visual inspection and LI, as well as to histopathologically assess the disease-free margin post-excision.

## Materials and Methods:

An observational study was carried out on 18 patients who presented with oral white lesions to a tertiary healthcare facility's Department of Oral and Maxillofacial Surgery. Documented informed consent was obtained. The inclusion criteria comprised leukoplakia, speckled leukoplakia, erosive lichen planus, carcinoma in situ (suspicious white lesion), and OSCC T1 and T2 lesions. The exclusion criteria included prior surgeries, chemotherapy or radiotherapy for head and neck cancer, adverse reactions to iodine, and patients diagnosed with hyperthyroidism. The lesion region was treated with 0.9% saline solution. The edges of the lesion, or its largest dimension, anteroposterior, supero-inferiorly, were measured with a calliper. Photographic documentation was also conducted. LI staining was conducted under local anaesthesia with adequate retraction, illumination, and support. The region received irrigation with 20 ml of LI, permitting no less than 30 seconds for staining to take place. Surplus fluid was extracted. The lesion site was given irrigation with 0.9% saline solution. The staining method was reiterated until satisfactory results were achieved. The border of the lesion, defined as its maximum anteroposterior and supero-inferior dimensions, was obtained with a calliper. Photographic documentation was successfully finalized. The lesion was excised, covering all accessible regions of non-stained mucosa. The removed tissue was sent for histological examination. The assessment criteria encompassed age, gender, habits, and lesion duration. The dimensions of the lesion were statistically assessed before and after the use of LI. The histopathological result was assessed for disease-free margins.

## Results:

The mean age of the study group was 40.11 years with a standard deviation of 13.06 years, ranging from 24 to 67 years. The sample consisted entirely of the male population (n=18). One patient was eliminated from the trial due to a serious reaction characterized by significant ulceration of buccal mucosa, which cleared within 7 days following the application of topical corticosteroids to the afflicted region. Table 1 (see PDF) presents an overview of the age distribution of participants. In the cohort of 18 individuals, the distribution of lesions was recorded as follows: 27.7% in the left buccal mucosa, 11.1% in the left buccal sulcus, 11.1% in the left retromolar region, 5.6% in the lower labial sulcus, 5.6% in the right angle of the mouth, 22.2% in the right buccal mucosa, and 11.1% in the right buccal sulcus. Table 2 (see PDF) provides a summary of the site dispersion. The excision margin was assessed both before and after each patient received LI. The anteroposterior border application ranged from 12 to 30 mm, with a mean ± SD of 19.33 ± 5.67. The superior-inferior margin ranged from 8 to 22 mm, with a mean ± SD of 15.17 ± 4.02. The range of the anteroposterior border post-application was 14 to 30 mm, with a mean ± SD of 20.78 ± 5.52. The superior-inferior border ranged from 9 to 24 mm, with a mean ± SD of 16.61 ± 4.06. The dimensional variation was 1.44 ± 0.76 antero-posteriorly and 1.44 ± 0.68 supero-inferiorly before and after the application, with a p-value of <0.001 for both antero-posterior and supero-inferior measurements. A summary of the margins is provided in Table 3 (see PDF). Dysplastic cells were identified in 11.1% of cases during the histological margin evaluation, while they were absent in 88.9% of cases. Table 4 (see PDF) provides an overview of the histopathological margin.

## Discussion:

The notion of a two-step mechanism in OC progression, characterized by the early emergence of a precursor OPMD that later evolves into OC, is recognized. Timely identification of oral mucosal epithelial dysplasia may prevent the advancement of these diseases into malignancy. The effective surgical management of oral cancer relies on the disease stage at diagnosis and the thorough excision of the tumour. Residual disease following surgical interventions is associated with rapid recurrence and an adverse outcome. The full removal of the lesion using adequate safe margins is therefore crucial [9]. LI is an excellent diagnostic adjuvant for identifying safe margins of OSCC, characterized by its simplicity, cost-effectiveness, non-invasiveness, rapid application, and high diagnostic accuracy [10, 11]. Noor *et al.* [12] conducted a comparable study to assess the diagnostic accuracy of LI staining in identifying the safe borders of OSCC, employing histopathology as the standard of excellence. The buccal mucosa was identified as the most prevalent site of occurrence in their investigation, after the tongue and lower vestibule. Other infrequent locations were the upper vestibule, lip, and maxillary sinus. The sensitivity and specificity of LI were 91% and 92.3%, respectively. The positive predictive value was 77%, while the negative predictive value was 92%. Our research indicates that the LI staining technique is effective for pinpointing the location of white lesions in the oral cavity. This study aligns with the findings of Yajima *et al.* [13], which indicate that iodine-unstained areas of epithelial dysplasia harbor cells that are nearly neoplastic and demonstrate heightened proliferation. Furthermore, it suggests that epithelial dysplasia located adjacent to squamous cell carcinoma should be surgically removed alongside the tumor. The management of enclosed oral epithelial dysplasia is essential for preventing oral cancer. Morikawa *et al.* [14] utilized fluorescence imaging and iodine solution to detect oral epithelial dysplasia and oral cancer. The integration of fluorescence vision with iodine solution is useful for delineating surgical margins in early tongue cancer. Sharma *et al.* [15] assessed the accuracy of *in vivo* staining using the double staining method of methylene blue and LI in contrast to methylene blue staining alone. Their study indicated that enhanced accuracy of the twofold staining method facilitates improved detection of dysplasia, significantly assisting clinicians in determining the nature of potentially malignant illnesses. McMahon *et al.* [18] performed a comparative analysis between an iodine-guided group and a white-light-guided control group. A significant difference between the two groups was observed only when all forms of dysplasia were categorized as positive, indicating that iodine-guided surgery is particularly effective in identifying moderate or mild dysplasia. Umeda *et al.* [16] reported similar outcomes and observed an absence of local recurrences in their single-arm study. De Koning *et al.* [17] demonstrated the efficacy of iodine in assessing mucosal safety margins, revealing that most margins are free from OSCC and dysplasia. The negative predictive value for OSCC and dysplasia in McMahon *et al.*'s iodine-guided surgery cohort indicates that iodine may effectively exclude moderate and mild dysplasia in the resection margin when compared to the results from the white-light-guided surgery group. Nagaraju *et al.* [18] established the diagnostic efficacy of Lugol's iodine in identifying premalignant and malignant lesions. The study demonstrated a sensitivity of 93%, specificity of 80%, positive predictive value of 98%, negative predictive value of 50%, and a diagnostic accuracy of 92% for the stain. The investigation demonstrated a sensitivity of 100%, with all patients showing positive staining for dysplastic changes. The delineation of dysplastic or malignant epithelium was established by an iodine-stained margin. A multicenter, randomized controlled study conducted by McCaul *et al.* demonstrated that the use of Lugol's iodine for visualizing margin dysplasia facilitates the complete excision of high-risk, precancerous mucosa during primary surgery, which may result in decreased local recurrence and enhanced survival rates [7]. Future research should involve larger samples and longitudinal designs to evaluate the progression of lesions in relation to the intensities and patterns of stain retention, thereby enabling more robust conclusions. One patient was eliminated from the trial due to an adverse reaction characterized by significant ulceration of buccal mucosa, which cleared within seven days following the application of topical corticosteroids to the afflicted region. This study was limited by a small sample group and employed a retrospective methodology. Secondly, the techniques employed to assess iodine solution were subjective, necessitating the incorporation of more objective metrics [19]. The examination of subjective and objective factors in OC screening is commencing [20, 21]. Consequently, additional prospective trials of OSCC screening and therapies for various oral sites are being organized.

## Conclusion:

A notable difference was detected following the application of Lugol's iodine to the lesions. Establishing surgical resection margins and delineating questionable sites for biopsies is advantageous. Lugol's iodine stain serves as a valuable tool in determining the potential advancement of suspicious lesions, therefore facilitating the early detection of oral cancer.
